# Trends of hypertensive disorders of pregnancy among the Medicaid population before and during COVID-19

**DOI:** 10.21203/rs.3.rs-3616259/v1

**Published:** 2023-11-29

**Authors:** Gang Hu, Jessica Lin, Heidi Feng, Ronald Horswell, San Chu, Yun Shen

**Affiliations:** Pennington Biomedical Research Center; Pennington Biomedical Research Center; Pennington Biomedical Research Center; Pennington Biomedical Research Center; Pennington Biomedical Research Center; Pennington Biomedical Research Center

## Abstract

Hypertensive disorders of pregnancy (HDP) are a group of high blood pressure disorders during pregnancy that are a leading cause of maternal and infant morbidity and mortality. The trend of HDP among the Medicaid population during the coronavirus disease of 2019 (COVID-19) is severely lacking. To determine the trends in the annual prevalence of HDP among Louisiana Medicaid pregnant women before and during the COVID-19 pandemic (2016–2021), a total of 113,776 pregnant women aged 15–50 years was included in this study. For multiparous individuals, only the first pregnancy was used in the analyses. Women with a diagnosis of each type-specific HDP were identified by using the ICD-10 codes. The prevalence of HDP increased from 10.5% in 2016 to 17.7% in 2021. The highest race/ethnicity-specific incidence of HDP was seen in African American women (13.1%), then white women (9.4%), followed by other women (7.9%). HDP remains as a very prevalent and significant global health issue, especially in African American women. Obesity and physical inactivity are major risk factors of HDP, which became amplified during the COVID-19 pandemic and led to a higher prevalence of HDP. Severe HDP substantially increases the risk of mortality in offspring and long-term issues in both the mother and infant. This is very pertinent to the Medicaid population due to the disparities and barriers that diminish the quality of healthcare they receive.

## INTRODUCTION

Hypertensive disorders of pregnancy (HDP) are a group of high blood pressure disorders during pregnancy that include gestational hypertension, chronic hypertension, preeclampsia, and preeclampsia superimposed on chronic hypertension^1,2^. Gestational hypertension is persistent de novo hypertension that arises at or after 20 weeks’ gestation in the absence of clinical features of preeclampsia. Chronic hypertension is high blood pressure (systolic blood pressure ≥ 140 mmHg or diastolic blood pressure ≥ 90 mmHg) predating the pregnancy or recognized before 20 weeks’ gestation^2^. Preeclampsia is gestational hypertension accompanied by ≥ 1 of the following new-onset conditions at or after 20 weeks’ gestation: protein in urine, and/or swelling in legs, feet, and hands^2^. Preeclampsia superimposed on chronic hypertension is chronic hypertension with new-onset proteinuria or other signs/symptoms of preeclampsia after 20 weeks’ gestation or chronic proteinuria with new onset hypertension^2^.

HDP complicates between 5–10% of all pregnancies and is a leading cause of maternal and infant morbidity and mortality^3–5^. In addition, HDP is a leading cause of maternal intensive care admissions^5^. Offspring of mothers with severe and early onset preeclampsia had more than six times the mortality risk compared to offspring of mothers with no HDP^6^. HDP is associated with aversive pregnancy and birth outcomes, such as preterm birth, low birth weight, small for gestational age, stillbirth, cesarean section, induced labor, admission to neonatal intensive care units (NICU), and perinatal mortality for the infant^7–9^.

Furthermore, mothers with HDP and their children have increased short-term and long-term risks for diseases and cardiometabolic disorders. Mothers with HDP have an increased risk of future cardiometabolic disease, such as hypertension, coronary heart disease, cardiac arrythmia, myocardial infarction, type 2 diabetes, and stroke^4,9^. In addition, HDP is associated with a 50% increased risk for cardiovascular disease (CVD) within five years after childbirth and a two to three times higher risk of hypertension between 2–7 years post-pregnancy^10^. Moreover, mothers with preeclampsia have an increased risk for CVD mortality as early as 10- years after childbirth.

Common risk factors associated with HDP are obesity, African American ethnicity, lower household income, lower educational level, advanced maternal age, rural residential area, lack of antenatal care, family history of hypertension, diabetes mellitus, sedentary life, and tobacco use^3,11–14^. Many studies have shown that African American women have a higher risk for HDP in the United States^1,9,15^. Several^16–18^, but not all studies^19,20^, show that women who tested positive for coronavirus disease of 2019 (COVID-19) during their pregnancy also had a greater risk of HDP.

The Louisiana population confronts many health challenges as a leading state in the prevalence of CVD, diabetes, obesity, physical inactivity, and tobacco use^21^. Louisiana has a higher rate in each of the major disease categories (heart disease and stroke, obesity, and diabetes) than the average rate of the United States^21^. In Louisiana, over 1.9 million people receive health care coverage through Medicaid, with many from low socioeconomic status (SES) households^22^. Medicaid provides medical benefits and services to individuals and families with low income, and those covered by Medicaid generally have poorer health and health outcomes. Medicaid enrollment was higher during the COVID-19 public health emergency than in previous years, partly due to the inability of Medicaid to disenroll members during that period. Several studies have revealed that low SES is associated with a higher risk of adverse pregnancy outcomes and a higher maternal mortality rate, which may be attributed to disparities in prenatal care^23,24^.

While several studies have assessed the trend of HDP, only a few studies have assessed the trend among the Medicaid population. This study aimed to determine the trends in the annual prevalence of HDP among Louisiana Medicaid pregnant women from 2016 to 2021 and to compare the prevalence of HDP before and during the COVID-19 pandemic.

## METHODS

The data source used in this study was from the Louisiana Medicaid program, which operates within the Louisiana Department of Health. The Louisiana Medicaid program covers approximately 38,000 pregnant women and newborn annually and links the dataset between the mother and child. The Centers for Medicaid Services (CMS) collects the Medicaid administrative data, which is derived from reimbursement information of payment of bills. This study will include Louisiana Medicaid datasets for all pregnancies between January 1, 2016, to December 31, 2021. The study and analysis plan were approved by both Pennington Biomedical Research Center and the Louisiana Department of Health Institutional Review Boards. We did not obtain informed consent from participants involved in our study because we used anonymized data compiled from electronic medical records.

Women aged 15–50 years during childbirth with a diagnosis of HDP were identified by using the ICD-10 codes. For multiparous individuals, only the first pregnancy was used in the analyses. Gestational hypertension is identified using the ICD-10 code O13 and specifically selecting women who were diagnosed at or after 20 weeks’ gestation. Chronic hypertension before pregnancy is identified by the ICD-10 code O10.01 and selecting women who had elevated blood pressure predating the pregnancy or recognized before 20 weeks’ gestation. Preeclampsia is identified by the ICD-10 code O14.0 and selecting women who were diagnosed at or after 20 weeks’ gestation. Preeclampsia superimposed on chronic hypertension is identified by the ICD-10 code O11.0 and selecting for women who were diagnosed before 20 weeks’ gestation. To determine which race/ethnicity to include in the race/ethnicity-specific analyses, the three largest race/ethnicity sample sizes were selected: white, Black, and other. The other races/ethnicities (Asian, Hawaiian or Pacific Islander, and Native American) were excluded in the race/ethnicity-specific analyses due to a smaller sample size (e.g., n = 1,685 for Asian women throughout the study period).

### Statistical Analysis

The number of pregnancies and prevalence for HDP overall and each type specific HDP of each study year and age group was reported. The General Linear Model was used to calculate the yearly age-specific prevalence of HDP and yearly race/ethnicity-specific prevalence of HDP. The linear trend in prevalence across time was tested using prevalence of total or type-specific HDP as the outcome variable and year as a continuous by using the Logistic regression. Direct standardization was used to calculate the age-standardized incidence to the 2010 Census population using the following age groups: 15–19, 20–24, 25–29, 30–34, 35–39, and 40–50. All statistical analyses are performed with SAS Software Version 9.4 (SAS Institute Inc.). The results were considered statistically significant when a two-sided P < 0.05.

## RESULTS

The number of first-time pregnancies for each age group and study year from 2016 to 2021 are shown in **Table 1**. Our study consisted of 113,776 first-time pregnant women. The average maternal age during childbirth was 26.4 ± 6.0 years and was not significantly different before and during the COVID-19 pandemic. The age group of 40–50 years had the smallest sample size, and the age group of 20–24 years had the largest sample size.

The annual prevalence of HDP for each age group was shown in **Table 2**. The crude incidence increased from 7.7% in 2016 to 13.2% in 2021, and the age-standardized rate increased from 10.5% in 2016 to 17.7% in 2021 (P for trend < 0.001). The highest prevalence of HDP for each study year was seen in the highest maternal age group, 40–50 years. The prevalence of HDP was higher in the years during the COVID-19 pandemic, with only one exception (age group of 35–39 years in 2020).

The annual prevalence of each type specific HDP for each age group was shown in **Table 3**. The crude incidence for gestational hypertension increased from 1.6% in 2016 to 2.3% in 2021, and the age-standardized rate had an overall increase of 2.0% in 2016 to 2.6% in 2021 (P for trend < 0.001). The crude incidence for chronic hypertension before pregnancy had an overall increase from 1.1% in 2016 to 1.9% in 2021, and the age-standardized rate had an overall increase from 2.1% in 2016 to 3.8% in 2021 (P for trend < 0.001). The crude incidence for preeclampsia increased from 3.6% in 2016 to 7.1% in 2021, and the age-standardized rate increased from 4.0% in 2016 to 7.9% in 2021. The crude incidence for chronic hypertension with superimposed preeclampsia had an overall increase from 1.3% in 2016 to 1.9% in 2021, and the age-standardized rate had an overall increase from 2.4% in 2016 to 3.8% in 2020 and then decreased to 3.3% in 2021 (P for trend < 0.001). The prevalence of each type specific HDP had an overall increase as the age group increased.

The annual race/ethnicity-specific incidence of HDP for each age group was shown in **Table 4**. The race/ethnicity-specific incidence of HDP has increased over the course of the study years, with the highest incidences seen during the COVID-19 pandemic. The mean maternal age during childbirth varied from 27.8 years in other women, to 26.2 years in African American women, and 25.9 years in white women. The age-standardized rate showed the highest incidence in African American women (19.2%), then white women (13.1%), and other women (10.7%) (P for trend < 0.001). The incidence of HDP showed the greatest increase across the study period in white women (5.9%), then African American women (5.3%) and women of other women (4.5%).

## DISCUSSION

### Main findings

In this present study, the age-standardized prevalence of HDP increased from 10.5% in 2016 to 17.7% in 2021. The trend seen in this study is in congruence with national and global data^1,25,26^. In Denmark, offspring of mothers with pre-eclampsia and eclampsia had an increased risk of all-cause mortality by 29% and 188%, respectively^6^. In this present study, the annual incidence of HDP increased for each race/ethnicity. The overall prevalence of HDP was also higher during the years of the COVID-19 pandemic compared to prior years.

The findings in our study showed the highest age-standardized rate and crude incidence of HDP during the COVID-19 pandemic. COVID-19 led to an inactive lifestyle with lockdown restrictions to limit direct contact between individuals and, therefore, reduce exposure to the virus^27^. Previous studies have attested to an increased risk of hypertension during COVID-19, with sedentary behavior as one of the principal risk factors for new-onset and worsening of hypertension^28,29^. A national survey reported that home confinement during the pandemic led the daily sitting time to increase from 5 to 8 hours (28.6%)^30^. Physical activity is also associated with a significantly reduced risk of gestational hypertensive disorders^31^. During the COVID-19 pandemic, fewer individuals met the physical activity guidelines (20% vs. 50%)^30^.

Moreover, a sedentary lifestyle due to the COVID-19 pandemic has led to an increase in obesity and overweightness^32,33^. In comparison to pregnant women with a lower body mass index (BMI), pregnant women with a higher BMI were 1.4 times more likely to develop preeclampsia/eclampsia^32^. This trend is in congruence with national studies^6,32,34^. The risk of preeclampsia in Latin American women increased by over three times in overweight and obese women^32^. Studies have shown an association between maternal overweightness/obesity before pregnancy and an increased risk of HDP among Chinese women and in sub-Saharan Africa^32,34^. A study in Denmark concluded that mothers with HDP and a history of diabetes, compared with offspring of mothers with HDP alone, had a 57% higher risk of infant mortality^6^.

Consistent with other studies^1,15^, the present study concluded that African American women had the highest prevalence of HDP. These racial and ethnic disparities may be attributed to differences in the quality of pre- and post-natal health care received, implicit biases, and racial microaggressions. In addition, African American women have a greater prevalence of maternal morbidity and cardiovascular comorbidities (obesity, chronic hypertension, and preexisting diabetes), which are all major risk factors for HDP^15^. Moreover, Minhas et al. showed that the morbidity ratio for African American women with HDP is disproportionately higher than white women with HDP (41.7 per 100,000 live births vs. 13.4 per 100,000 live births)^15^.

Another prominent risk factor of HDP is a low SES, with the highest HDP mortality rate seen in areas of poverty^25^. Many individuals with a low SES are a part of the Medicaid population. The mortality rate of HDP is inversely correlated with low total family incomes, with a significant aspect due to the role of economic support in prenatal healthcare for both pregnant women and fetuses^25^. Many members of the Medicaid population have less access to quality health care and encounter numerous obstacles and challenges, such as health disparities due to social determinants of health, a lack of transportation to their medical appointments, and unaffordability of care due to a lack of insurance coverage. In addition, their quality of care pre-and post-natal periods may be decreased due to unawareness of critical information, sociodemographic factors (e.g., level of education, marital status), and the rising cost of health care^35^. Moreover, mothers with HDP and a low education level had a 49% higher risk of all-cause infant mortality compared to mothers with HDP alone^6^. Thus, the Medicaid population is exceedingly vulnerable to HDP.

## STRENGTHS AND LIMITATIONS

The main strength of this present study is that a large cohort of pregnancies were obtained in the Louisiana Medicaid Program dataset to assess the trend in the incidence of HDP in the Louisiana Medicaid population. In addition, our dataset had a large sample size of African American and white women with available information on each type-specific HDP. However, some limitations to our findings are that they pertained to the Louisiana Medicaid population and may not translate well to the non-Medicaid population. Many risk factors are more prominent in the Medicaid population, which may explain why there are discrepancies in the morbidity and mortality rate of diseases between the Medicaid population and the non-Medicaid population. Another limitation is that the Medicaid dataset may contain biased estimates because it is difficult to quantify an accurate representation of individuals with chronic hypertension. Many individuals may remain unaware of their condition or do not have the means to continue to monitor it with weekly/monthly appointments.

## CONCLUSION

HDP remains a very prevalent and significant global health issue, especially in African American women and during the COVID-19 pandemic. Severe HDP substantially increases the risk of mortality in offspring and long-term issues in both the mother and infant. Therefore, an emphasis on preventative measures is crucial, such as increasing the quality of prenatal care, clinical evaluation, and counseling for better health outcomes, providing economic support (for low-income mothers), and raising more awareness for management of HDP. This is very pertinent to the Medicaid population due to the disparities and barriers that diminish the quality of healthcare they receive. Future studies can determine if there are any racial disparities present in the types of care women receive prenatally when they are at risk of HDP and how those disparities may affect both the short-term and long-term health of the infant.

## Data Availability

The datasets generated and analysed during the current study are available from the corresponding author on reasonable request.
